# Friesian horses as a possible model for human acquired aortopulmonary fistulation

**DOI:** 10.1186/s13104-016-2201-5

**Published:** 2016-08-15

**Authors:** V. Saey, T. Vandecasteele, G. van Loon, P. Cornillie, M. Ploeg, C. Delesalle, A. Gröne, I. Gielen, R. Ducatelle, K. Chiers

**Affiliations:** 1 Department of Pathology, Bacteriology and Poultry Diseases, Ghent University, Merelbeke, Belgium; 2 Department of Morphology, Ghent University, Merelbeke, Belgium; 3 Department of Large Animal Internal Medicine, Ghent University, Merelbeke, Belgium; 4 Department of Pathobiology, Faculty of Veterinary Medicine, Utrecht University, Utrecht, The Netherlands; 5 Department of Comparative Physiology and Biometrics, Ghent University, Merelbeke, Belgium; 6 Department of Veterinary Medical Imaging and Small Animal Orthopaedics, Faculty of Veterinary Medicine, Ghent University, Merelbeke, Belgium

**Keywords:** Aortic rupture, Pseudoaneurysm, Aortopulmonary fistulation, Friesian horses

## Abstract

**Background:**

Acquired aortopulmonary fistulation is a rare condition in humans. It usually results as a late complication of a true or pseudoaneurysm of the thoracic aorta. It is most commonly associated with trauma or surgery, less commonly with atherosclerosis, inflammation, hypertension or Marfan’s syndrome. Aortopulmonary fistulation is also seen as a rare complication of acute aortic dissection. On rare occasions, acquired aortopulmonary fistulation is reported in aged patients without any of the above mentioned triggering factors. Thus, these cases should be considered as idiopathic aortopulmonary fistulation. Clearly, the pathogenesis of this condition is not yet completely understood. Friesian horses are highly inbred and are affected by several genetic conditions. Rupture of the thoracic aorta has a relatively high prevalence in Friesian horses and is often characterized by the formation of a pseudoaneurysm with subsequent fistulation into the pulmonary artery. Affected animals may survive for several weeks to months.

**Findings:**

Here we performed vascular casting in three affected Friesian horses. In all three cases, an aortic rupture at the caudoventral side of the aorta was connected with a rupture of the main pulmonary artery just proximal to its bifurcation.

**Conclusions:**

Affected Friesians show a consistent location and configuration of the aortic rupture site, very similar to the human condition and therefore could act as a spontaneous model to study this disease.

**Electronic supplementary material:**

The online version of this article (doi:10.1186/s13104-016-2201-5) contains supplementary material, which is available to authorized users.

## Background

In spite of the relative frequency of thoracic aortic (pseudo)aneurysms and the close anatomical relation between aorta and truncus pulmonalis, aortopulmonary fistulation is very rare in humans. In a review by Boyd [1], 4000 cases of thoracic aortic aneurysms were studied and only in 4 % there was an aortopulmonary fistulation. The diagnosis is made primarily by echocardiography and aortography [2], followed by further imaging, such as computed tomography (CT) and magnetic resonance imaging (MRI) [3]. Reports of successful surgical management of aortopulmonary fistulas are scarce due to the magnitude of operative problems encountered [4–6].

**Fig. 1:**
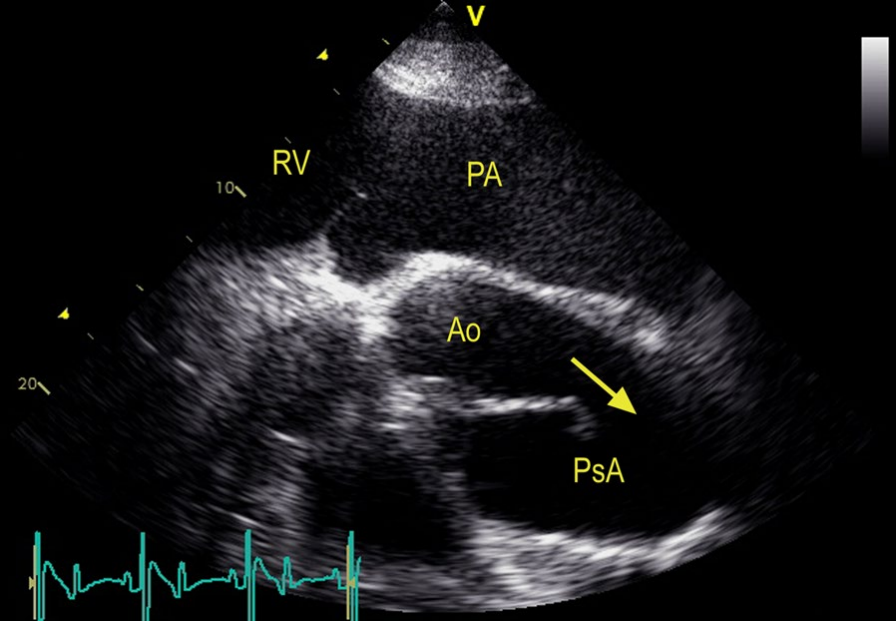
Ultrasound image showing the blood flow (*arrow*) from the ruptured aorta (Ao) into the pseudoaneurysm (PsA). The pulmonary artery (PA) is severely dilated due to pulmonary hypertension. (*RV* right ventricle)

**Fig. 2:**
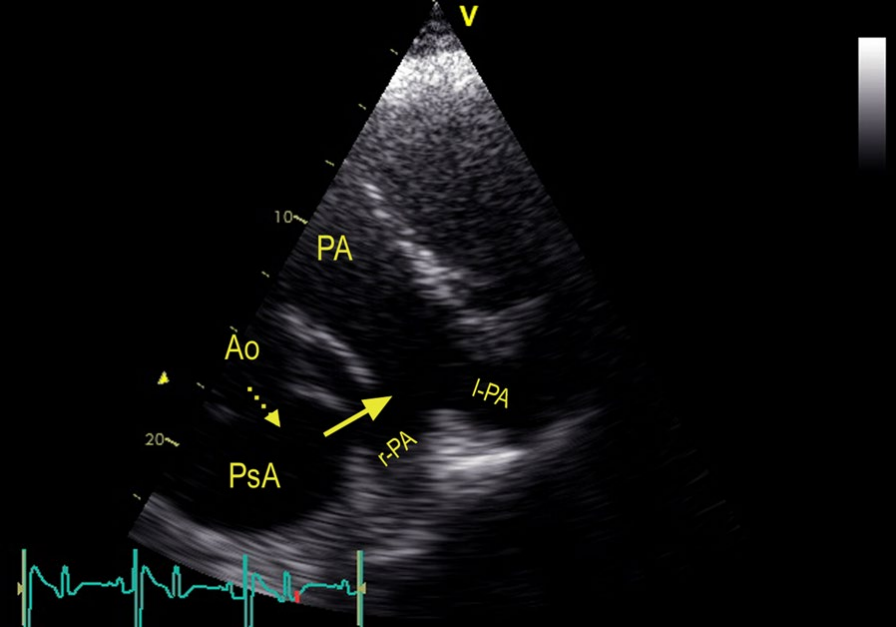
Ultrasound image: from the ruptured aorta (Ao) blood flows (*dotted arrow*) into the pseudoaneurysm (PsA) and subsequently through the fistula that enters the pulmonary artery (PA) near the bifurcation towards the left (l-PA) and right (r-PA) branch of the PA

Aortic rupture is an extremely rare condition in the general horse population. It can occasionally be seen in older breeding stallions [7] and sports horses in full exercise [8]. In Friesian horses, however, aortic rupture is relatively common. We have demonstrated that it typically occurs as a transverse tear located immediately proximal to the ligamentum arteriosum, the remnant of the ductus arteriosus [9, 10]. The ductus arteriosus shunts approximately 2/3 of the fetal blood directly to the aorta, thus bypassing the pulmonary artery [11]. Between 2007 and 2013, we have diagnosed 37 Friesian horses with aortic rupture during postmortem examination at the Faculty of Veterinary Medicine of Ghent University, Belgium, the Faculty of Veterinary Medicine of Utrecht University, and Wolvega Equine Clinic, the Netherlands. Of these 37 affected Friesians, 27 were diagnosed with an aortopulmonary fistula with survival for several weeks to months [10]. Three dimensional visualization of the fistulation between the aorta and pulmonary artery at necropsy is difficult due to extensive pseudoaneurysm formation, sometimes combined with dissections and periaortic hematomas [9]. Also in vivo visualization of this structure is hard to obtain since medical imaging is restricted to cardiac ultrasound due the large posture of horses [12] (Figs. 1, 2). Recently we reported on the potential benefits of performance of transesophageal ultrasound in attained cases to gain a better view on this region [13].

## Methods

To obtain a better insight into the 3D conformation of the site of rupture, post-mortem vascular casting of the aortopulmonary fistulation was performed in three affected Friesian horses (horse 1: mare, 4 years; horse 2: gelding, 11 years; horse 3: mare, 6 years) using the technique described by Vandecasteele et al. [14]. Silicone casting of the proximal thoracic aorta and pulmonary artery was done in the first two horses. In the third horse, a Technovit® 7143 cast was made. The complete cardiopulmonary set with the intact vessels was dissected from the thorax. The heart was then positioned in an upside down position. The left and right ventricle were opened and the aorta and truncus pulmonalis were flushed with a hosepipe. The silicone (base and catalyst, ratio 1:1) or Technovit® 7143 was then infused through a funnel in the left and right ventricle into the aorta and truncus pulmonalis. After hardening overnight at room temperature, the casts were dissected.

## Results

**Fig. 3:**
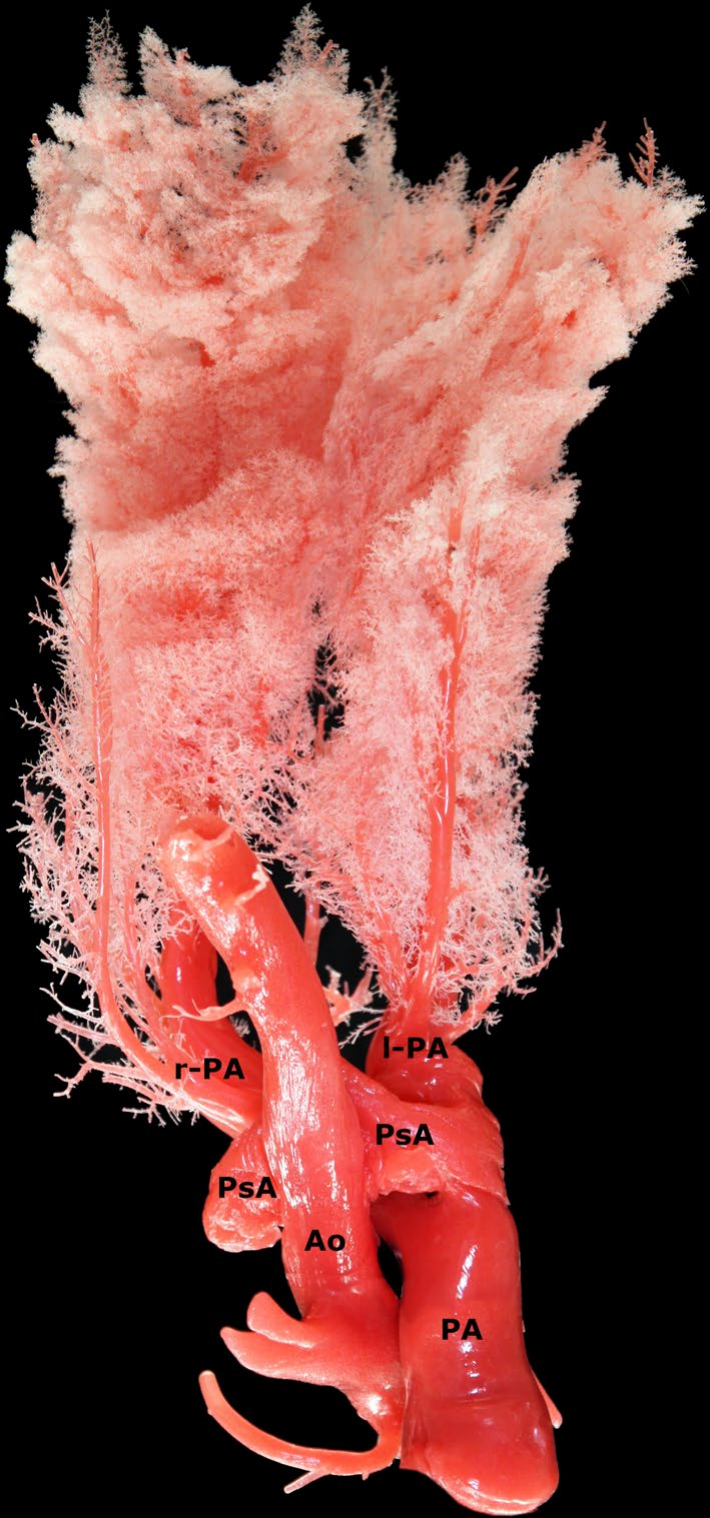
Dorsal view of a silicone cast from a Friesian horse with the caudoventral aortic rupture (Ao) and aortic pseudoaneurysm (PsA) fistulating into the dorsal side of the pulmonary artery (PA). (l-PA: left branch of the pulmonary artery, r-PA: right branch of the pulmonary artery)

Vascular casting in all three samples revealed an aortic rupture at the caudoventral side of the aorta and a rupture of the main pulmonary artery just proximal to its bifurcation (Fig. 3). The fistula between both arteries consisted of a pseudoaneurysm containing several (1-3) pocket-like spaces. These findings were consistent with the post-mortem findings described earlier [9]. An illustration of the technovit cast was included as Additional file 1.

## Discussion

Pseudoaneurysms result from disruption of all three layers of the arterial wall with the formation of a bloodfilled cavity lined by endothelium and supported by granulation, adventitial and periadventitial tissues [15]. True aortic aneurysms are expansions of the entire vessel wall, caused by segmental weakening of the wall due to a primary or secondary defect in the matrix structures. Loss of elastin has long been considered the hallmark of aneurysm formation, but it is now accepted that impaired collagen homeostasis is the main cause [16]. Aortic aneurysms are usually seen abdominally in older people. Hypertension, smoking and hypercholesterolemia are predisposing factors [17]. Thoracic aneurysms are mainly found in young people however and generally occur due to an inherited connective tissue disorder [17, 18]. Even apart from genetic disorders of collagen and elastin, a familial predisposition has been shown as well [19]. Rupture of the thoracic aorta in Friesian horses is not associated with the formation of a true aneurysm. Pseudoaneurysm formation is however typical [9, 10].

In humans, in more than 90 % of the cases, pseudoaneurysms typically occur at the aortic isthmus (between the left subclavian and the third intercostal artery) near the ligamentum arteriosum [20, 21]. Pseudoaneurysms are mainly associated with accidents involving pronounced deceleration or torsional trauma [21]. The exact pathophysiology of aortic pseudoaneurysms formation induced by blunt traumatic injury is still unknown, but it is most likely the result of a complex interaction between both motion of anatomical structures and local force loading [22]. Approximately 2 % of human patients surviving blunt traumatic aortic rupture live long enough to develop chronic pseudoaneurysms and periaortic hematomas [21, 23]. The periadventitial aortic isthmus tissue at that site seems to offer protection against complete aortic transection with subsequent acute exsanguination [21]. Aortopulmonary fistulation in humans can develop even decades after the traumatic event [24]. It is striking, that both pseudoaneurysms and periaortic hematomas occur at the same location in Friesian horses as in humans suffering from traumatic injury. This could indicate a common pathogenic mechanism predisposing certain humans for aortopulmonary fistulation after traumatic aortic injury. However, in contrast to humans, none of the affected Friesian horses had a history or showed signs of traumatic injury. Furthermore, inflammation and atherosclerosis, two other predisposing factors for pseudoaneurysm formation in humans [15], were not reported in Friesians [10].

It is believed that the aortopulmonary fistulation develops by continuous pulsatile friction between the wall of the aortic aneurysm or pseudoaneurysm and the pulmonary artery [25]. The aortic arch loops over the left pulmonary artery and the bifurcation of the main pulmonary trunk, to which it remains connected by the ligamentum arteriosum [26]. Also in horses, the thoracic aorta is positioned closely to the left pulmonary artery at the level of the bifurcation [27]. However, the geometry of the equine aorta differs from humans as the typical deviation of the aortic arch in humans is almost absent in horses [28]. In Friesian horses with an aortopulmonary fistulation, the pseudoaneurysm typically merges just proximal to the bifurcation of the pulmonary artery. In humans, fistulation into the pulmonary artery is mainly observed when the aortic intimal tear is present at the left side [29]. The latter is however rare, since aortic intimal tear formation usually occurs at the right anterolateral side of the ascending aortic wall which is the area that endures the highest stress [30]. This could explain the discrepancy between the relative high frequency of chronic aortic (pseudo)aneurysms and the rare incidence of APF.

It is worth mentioning that in all Friesians horses with an aortopulmonary fistulation, a patent ductus arteriosus was excluded as an intact scar of the former ligamentum arteriosum could be noticed. In horses, the ductus arteriosus closes physiologically within 3 days after birth [31] and the incidence of patent ductus arteriosus is very low [32].

Histologic findings in aortic rupture and aorto-pulmonary fistulation in 20 affected Friesians included accumulation of mucoid material, disorganization and fragmentation of elastic laminae, hypertrophy of smooth muscle cells and medial necrosis of the aortic wall. Inflammation was minimal [10]. Recently it was shown that collagen metabolism differs between Friesian and Warmblood horses [33]. It is feasible that a genetic disorder superimposed upon this aberrant Friesian connective tissue disorder is the cause of aortic rupture and aortopulmonary fistulation in this breed. It is feasible that a genetic disorder superimposed upon this aberrant Friesian connective tissue turn-over is the cause of aortic rupture and aortopulmonary fistulation in this breed. Biomechanical differences could not be detected in the thoracic aorta between Friesian horses with aortic rupture and nonaffected horses. With that respect, a possible scenario is the existence of a local, hereditary defect in the aortic wall, rather than a generalized aortic disease [34].

Reports in literature of aortopulmonary fistulation in humans and description of clinical symptoms are confusing as the term aortopulmonary fistula is used not only to describe fistulas terminating in the pulmonary artery, but also those terminating in the bronchial tree [35–37]. However, the latter should be called aortobronchial fistulas [38]. Chest pain, intermittent or recurrent hemoptysis, dyspnea and other respiratory symptoms have been mentioned as typical symptoms of aortopulmonary fistulation [39, 40]. Indeed, hemoptysis mainly occurs when the pseudoaneurysm “leaks” into the bronchopulmonary tree, thus in case of an aortobronchial fistula [41]. However, an aortopulmonary fistulation in humans can also result in massive hemoptysis, especially if pulmonary thrombo-embolism occurs [41], but this is not yet described in Friesian horses. A common finding in aortopulmonary fistulation in both humans and Friesian horses is high-output cardiac failure [6, 25, 41, 42]. Friesians with an aortopulmonary fistulation typically suffer from tachycardia, pulmonary edema, ventral edema, mild fever, colic and a bounding arterial pulse [9]. Due to the dimensions of the connection in Friesians, there is no treatment available at this moment [39] and the disease process will always end fatal once clinical signs develop.

In summary, the aortic rupture and aortopulmonary fistulation formation in Friesian horses occurs without any history of trauma or signs of inflammation. Considering the similar location of the lesions in Friesian horses and humans, the chronic aspect of this disease, the fatal outcome and the possibility to obtain vascular casts, the Friesian horse could be a valuable spontaneous model for this condition in humans.

## Additional file

10.1186/s13104-016-2201-5 Rotating pdf of the technovit cast showing the aortopulmonary connection in an affected Friesian horse.

## Abbreviations

CT: computed tomography
MRI: magnetic resonance imaging

## References

[1] Boyd LJ (1924). A study of four thousand reported cases of aneurysm of the thoracic aorta. Am J Med Sci.

[2] Razzouk A, Gundry S, Wang N, Heyner R, Sciolaro C, Van Arsdell G, Bansal R, Vyhmeister E, Bailey L (1993). Pseudoaneurysms of the aorta after cardiac surgery or chest trauma. Am Surg.

[3] Kort S, Tunick PA, Applebaum RM, Hayes R, Krinsky GA, Sadler W (2001). Acquired aortopulmonary artery fistula: diagnosis by multiple imaging modalities. J Am Soc Echocardiogr.

[4] Lahey SJ (1993). Successful surgical management of an aortic arch aneurysm with acute aorto-pulmonary fistula. Ann Thorac Surg.

[5] Coselli S, LeMaire SA, van Gleve RD (1995). Rupture of a dissecting thoracic aortic aneurysm into the pulmonary artery: successful surgical repair. Cardiovasc Surg.

[6] Atay Y, Can L (1998). Yagdi, Buket S. Aortopulmonary artery fistula. Presenting with congestive heart failure in a patient with aortic dissection. Tex Heart Inst J.

[7] Rooney JR, Prickett ME, Crowe MW (1967). Aortic ring rupture in stallions. Pathol Vet..

[8] Brown CM, Kaneene JB, Taylor RF (1988). Sudden and unexpected death in horses and ponies: an analysis of 200 cases. Equine Vet J.

[9] Ploeg M, Saey V, de Bruijn CM, Grone A, Chiers K, van Loon G, Ducatelle R, van Weeren PR, Back W, Delesalle C (2013). Aortic rupture and aorto-pulmonary fistulation in the Friesian horse: characterisation of the clinical and gross post mortem findings in 24 cases. Equine Vet J.

[10] Ploeg M, Saey V, Delesalle C, Grone A, Ducatelle R, de Bruijn CM, Back W, van Weeren PR, van Loon G, Chiers K (2015). Thoracic aortic rupture and aortopulmonary fistulation in the Friesian horse: histomorphologic characterization. Vet Pathol.

[11] Bankl H (1977). Congenital malformations of the heart and great vessels: synopsis of pathology, embryology and natural history.

[12] van Loon G, De Clercq D, De Bruijn CM, Decloedt A, Verheyen T, Saey V, Ducatelle R, Chiers K, Ploeg M, Back W, Delesalle C, Deprez, P. Aortic rupture and aortopulmonary fistulation: increased prevalence in Friesian horses and importance of early ante-mortem diagnosis. Conference Abstract Association Vétérinaire Equine Française (AVEF). 2011.

[13] De Bruijn CM, van Loon G, Ploeg M, Gröne A, De Clercq D, Decloedt A, Van Weeren PR, Back W, Delesalle C (2013). Use of transoesophageal ultrasound to visualise the aortopulmonary region in two normal friesian horses and three friesians with aortic rupture or aortopulmonary fistulation. Equine Vet J.

[14] Vandecasteele T, Vandevelde K, Doom M, Van Mulken E, Simoens P, Cornillie P (2015). The pulmonary veins of the pig as an anatomical model for the development of a new treatment for atrial fibrillation. Anat Histol Embryol.

[15] Rajiah P (2013). CT and MRI in the evaluation of thoracic aortic diseases. Int J Vasc Med..

[16] Thompson RW, Geraghty PJ, Lee JK (2002). Abdominal aortic aneurysms: basic mechanisms and clinical implications. Curr Probl Surg.

[17] Jain D, Dietz HC, Oswald GL, Maleszewski JJ, Halushka MK (2011). Causes and histopathology of ascending aortic disease in children and young adults. Cardiovasc Pathol..

[18] Reed D, Reed C, Stemmermann G, Hayashi T (1992). Are aortic aneurysms caused by atherosclerosis?. Circulation.

[19] Elefteriades JA (2002). Natural history of thoracic aortic aneurysms: indications for surgery, and surgical versus nonsurgical risks. Ann Thorac Surg.

[20] Nzewi O, Slight RD, Zamvar V (2006). Management of blunt thoracic aortic injury. Eur J Vasc Endovasc Surg.

[21] Gleason TG, Bavaria JE, Cohn LH, Edmunds LHJ (2003). Trauma to great vessels. Cardiac surgery in the adult.

[22] Pearson R, Philips N, Hancock R, Hashim S, Field M, Richens D, McNally D (2008). Regional wall mechanics and blunt traumatic aortic rupture at the isthmus. Eur J Cardiothorac Surg.

[23] Katsumata T, Shinfeld A, Westaby S (1998). Operation for chronic traumatic aortic aneurysm: when and how?. Ann Thorac Surg.

[24] Giglioli C, Cecchi E, Angelotti P, Venditti F, Calabretta R, Scheggi V, Alterini B, Stefano P (2013). Aortopulmonary fistula presenting with right ventricular dysfunction following blunt chest trauma. J Card Surg.

[25] Bol A, Missault L, Dossche KM, Delanote J (2006). Aortopulmonary artery fistula in atherosclerotic pseudoaneurysm presenting with congestive heart failure after aortic valve replacement. Acta Chir Belg.

[26] Drake RL, Vogl WA, Mitchell AWM (2010). Gray’s anatomy for students.

[27] Sisson S, Grossman JD (1938). The anatomy of domestic animals.

[28] Casteleyn C, Cornillie P, Simoens P (2010). Anatomie comparée de l’arc aortique de l’homme, des animaux domestiques et des animaux de laboratoire: à la recherche d’un modèle, pour faciliter l’étude des maladies vasculaires humaines. STAL.

[29] Massetti M, Babatasi G, Rossi A, Kapadia N, Neri E, Bhoyroo S, Gerard JL, Commeau P, Khayat A (1995). Aortopulmonary fistula: an uncommon complication in dystrophic aortic aneurysm. Ann Thorac Surg.

[30] Bauer M, Siniawski H, Pasic M, Schaumann B, Hetzer R (2006). Different hemodynamic stress of the ascending aorta wall in patients with bicuspid and tricuspid aortic valve. J Card Surg.

[31] Machida N, Yasuda J, Too K, Kudo N (1988). A morphological study on the obliteration processes of the ductus arteriosus in the horse. Equine Vet J.

[32] Guarda I, Schifferli CA, Alvarez LH, Probst A, König HE (2005). Ductus arteriosus persistens bei einem fohl. Vet Med Austria.

[33] Saey V, Ploeg M, Delesalle C, van Loon G, Gröne A, Ducatelle R, Duchateau L, Chiers K (2016). Morphometric properties of the thoracic aorta of warmblood and Friesian horses with and without aortic rupture. J Comp Pathol.

[34] Saey V, Famaey N, Smoljkic M, Claeys E, van Loon G, Ducatelle R, Ploeg M, Delesalle C, Grone A, Duchateau L, Chiers K (2015). Biomechanical and biochemical properties of the thoracic aorta in warmblood horses, Friesian horses, and Friesians with aortic rupture. BMC Vet Res..

[35] Hope MD, Hope TA, Urbania TH, Higgins CB (2010). Four-dimensional flow magnetic resonance imaging with wall shear stress analysis before and after repair of aortopulmonary fistula. Circ Cardiovasc Imaging.

[36] Hampson S, Pepper J (1987). Aortopulmonary fistula: role of computed tomography. Thorax.

[37] Sabety AM, Gerard FP (1966). Aorto-pulmonary fistula: report of a case. Chest J.

[38] Ahmadi ZH, Ansari AZ, Saghebi SR, Kianfar AA, Hashemian SM, Kahkouee S, Naderi H (2014). Massive hemoptysis, a presentation of invasion of aneurysm of descending aorta to bronchopulmonary tree. Arch Iran Med..

[39] van Loon G, Verheyen T, Decloedt A, Delesalle C, Schauvliege S, De Wolf D. Use of a transcatheter occlusion device in a 9-year old Friesian gelding with aortopulmonary fistula. Eur Vet Conf. Voorjaarsdagen; 2011:365.

[40] Killen DA, Muehlebach GF, Wathanacharoen S (2000). Aortopulmonary fistula. South Med J.

[41] Belgi A, Altekin E, Yalcinkaya S, Tuzuner FE (2003). Acquired aorto-pulmonary fistula: a case of ruptured aneurysm of the thoracic aorta. Anadolu Kardiyol Derg.

[42] Dixit MD, Gan M, Narendra NG, Mohapatra RL, Halkatti PC, Bhaskar BV (2009). Aortopulmonary fistula: a rare complication of an aortic aneurysm. Tex Heart Inst J.

